# Stimulator-multiplexing framework of microwave-infrared compatible reconfigurable metasurface integrated with LED array

**DOI:** 10.1515/nanoph-2025-0013

**Published:** 2025-03-19

**Authors:** Yuxi Li, Ruichao Zhu, Sai Sui, Yina Cui, Yuxiang Jia, Yajuan Han, Xinmin Fu, Cunqian Feng, Shaobo Qu, Jiafu Wang

**Affiliations:** AeroSpace MetaMaterials Laboratory of Suzhou National Laboratory, 66488 Air Force Engineering University, Xi’an, 710038, China; Shaanxi Province Key Laboratory of Artificially-Structured Functional Materials and Devices, Air Force Engineering University, Xi’an, 710038, China; Air and Missile Defense College, Air Force Engineering University, 710038, Xi’an, Shaanxi, China

**Keywords:** reconfigurable metasurface, stimulator-multiplexing, reconfigurable characteristics, microwave band, infrared band

## Abstract

Metasurface can accurately control and manipulate electromagnetic (EM) waves with high degree of freedom, which is mainly due to their subwavelength structures and functional arrangements. However, most reconfigurable metasurfaces are currently limited to modulating EM waves in a single band. In order to further expand the application scenarios of metasurface, a stimulator-multiplexing framework of microwave-infrared compatible reconfigurable metasurface integrated with LED array is proposed. In this framework, a photoresistor is fully embedded into the meta-atom as an active device. Its resistance value can be adjusted through controlling the luminous intensity of the LED array. The LED array generates excitation light source, along with infrared characteristics. Therefore, it is not only the controller in the microwave band, but also the basic pixel in the infrared band. The framework adopts the way of stimulator-multiplexing, and the reconfigurable characteristics in the microwave and infrared bands can be realized through a single meta-atom structure. This work greatly enriches the metasurface design, which has a wide application prospect in many fields such as information transmission, and adaptive intelligent perception.

## 1 Introduction

Metasurface is an artificial material composed of sub-wavelength structures arranged on a two-dimensional surface in a periodic or non-periodic manner [1], [2], [3], [4], [5], [6]. Through accurately designing the geometric shape and spatial arrangement, these artificial structures can flexibly regulate the characteristics of EM wave amplitude, phase and polarization mode [7], [8], [9]. Metasurface has developed rapidly because it is very compatible with current integrated circuit processes, and has the advantages of small size, thin thickness, low loss, simple manufacturing and suitable for miniaturization [10], [11]. Metasurface has a strong ability to manipulate EM waves in visible [12], [13], [14], [15], infrared [16], terahertz [17], [18], [19], and microwave [20], [21], [22] bands. At present, it is widely used in many fields such as holographic imaging [23], [24], [25], metalenses [26], [27], [28], polarization conversion [29], [30], [31], and EM absorption [32], with unexpected effects.

However, most metasurfaces can only adjust EM wave in a single band, without meeting the requirements of complex EM environment [33], [34], [35]. In recent studies, multilayer structure metasurface realize EM modulation in the microwave and infrared bands. They are now widely used in the field of camouflage [36]. Due to the different principles of camouflage technology, the corresponding materials, structures and design paradigms will be different [37], [38]. Radar camouflage materials need to have the characteristics of high absorption rate and low reflectivity, which can reduce the energy of reflected waves [39], [40], [41]. In contrast, infrared camouflage materials need to have low infrared emission in order to achieve infrared stealth [42], [43], [44], [45]. According to Kirchhoff’s law, low emissivity means low absorption rate [46]. This multilayer structure metasurface can achieve both high microwave absorption and low infrared emission characteristics. However, this metasurface is not reconfigurable, which greatly limits the application scope.

**Figure 1:**
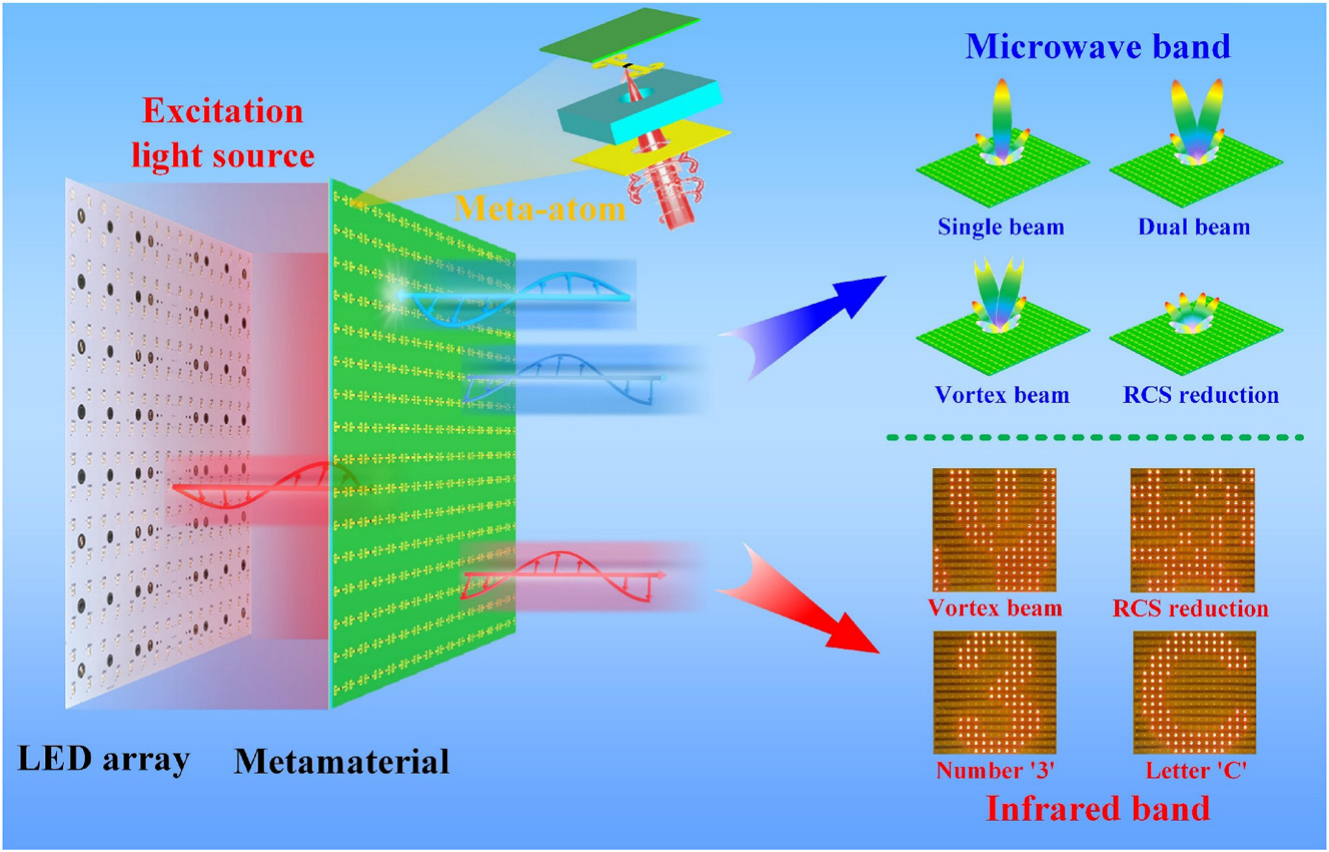
Schematic diagram of the principle of microwave-infrared compatible RM and its application.

Based on the above research status, this paper proposes a stimulator-multiplexing framework for microwave-infrared compatible reconfigurable metasurface (RM) based on LED array, as shown in Figure 1. As an active device, the photoresistor is embedded in the meta-atom and adjusted by the LED array. The lamp beads correspond to the meta-atoms one-to-one, so the photoresistor value can be changed individually. Through designing phase distributions, metasurface realizes different EM functions such as single beam, dual beam, vortex beam, and RCS reduction. The framework can dynamically regulate EM wave response in the microwave band. The LED array excites the light source to result in the increase of temperature, which is accompanied by infrared characteristics. Different coding patterns are designed to control the on-off of lamp beads. Therefore, the framework can also realize reconfigurable characteristic in the infrared band. This work enriches the metasurface design, and realizes reconfigurable characteristics in the microwave and infrared bands with a single structure. More importantly, the framework adopts the dynamic control mode of direct light control. Compared with the traditional control methods, the control mode has the advantages of lower system complexity, higher integration, lower energy consumption and more flexible control, which provides a new idea for the development of intelligent metasurface. It has potential application prospects in the fields of information transmission, and adaptive intelligent perception.

## 2 Meta-atom structure design and EM response

**Figure 2:**
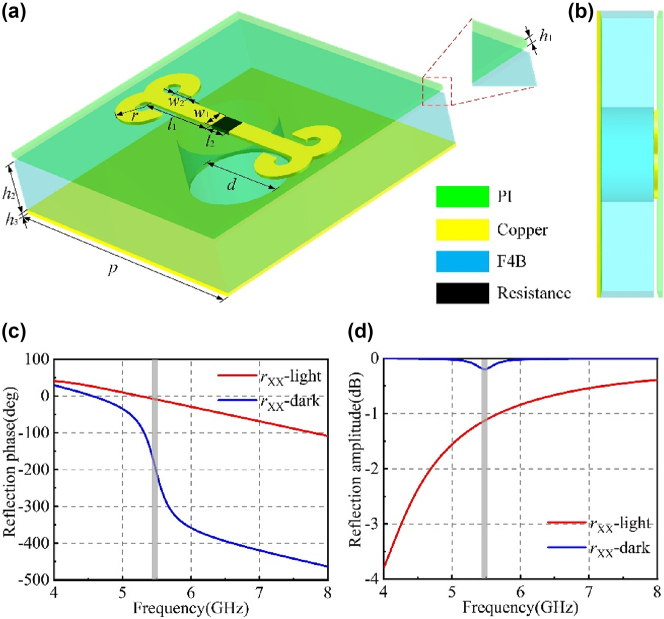
Dynamic meta-atom: (a–b) structure diagram and geometric parameters; (c) reflection phase in the ‘0’ and ‘1’ states; (d) reflection amplitude in the ‘0’ and ‘1’ states.

In this paper, a dynamic meta-atom structure containing a GT36516 photoresistor is proposed, as shown in Figure 2a and b, which is composed of four layers. The top structure is a transparent PI film (dielectric constant *ɛ*_*r*_ = 3.4, loss tangent tan *δ* = 0.001), which is used to achieve mechanical support for the metal structure. Moreover, this material can also ensure that the light emitted by the LED array is transmitted. The metal structure layer consists of four metal split-ring resonators and a metal wire. A photoresistor is loaded in the middle of the metal wire to adjust the resonance characteristics of the meta-atom. The other two layers are F4B dielectric substrate (dielectric constant *ɛ*_*r*_ = 2.65, loss tangent tan *δ* = 0.001) and metal reflective backboard. The metal reflective backboard is used to reflect EM wave. In order to ensure that the light emitted can adjust the photoresistor value, a hole is punched at the position corresponding to the photoresistor on the dielectric substrate and the reflective backboard, as shown in Figure 2a.

The judiciously designed meta-atom parameters are designed as follows. The meta-atom structure period is *p* = 16 mm. The thickness of the PI film is *h*_1_ = 0.05 mm, thickness of the F4B dielectric substrate is *h*_2_ = 3 mm, and thickness of the metal reflective backplate is *h*_3_ = 0.035 mm. The length of the metal wire is *l*_1_ = 4.8 mm and width is *w*_1_ = 1 mm. The width of the opening gap is *l*_2_ = 1.4 mm. The outer diameter of the split-ring resonator is *r* = 1.5 mm, width is *w*_2_ = 1 mm, and opening angle is 90°. The hole diameter is *d* = 5.4 mm.

The photoresistor value can reach 0.3 MΩ in the dark environment, which is in an open state. The metal structure is truncated into two parts. With the gradual increase of light intensity, it gradually decreases to tens of ohms, which is in a conductive state. The metal structure is connected into a whole. Codes ‘0’ and ‘1’ represent the photoresistor state in the dark environment or the light environment, respectively. After the photoresistor is packaged, it generates a certain capacitance and inductance.

First, the photoresistor values corresponding to the codes ‘0’ and ‘1’ are determined. The EM responses of the judiciously designed meta-atom are precisely simulated with the assistance of the computer simulation technology CST Microwave Studio. A large number of data simulations are carried out through changing the photoresistor value. The EM response under different resistance values is shown in Figure S1. According to the simulation results, when the frequency is 5.47 GHz, the reflection phase difference between 50 Ω and 0.3 MΩ is π, and the reflection amplitude is almost equal, which meets the conditions of phase modulation of the meta-atom. The specific results are shown in Figure 2c and d. Supplementary information Note S1 for more details on the EM response under different resistance values.

**Figure 3:**
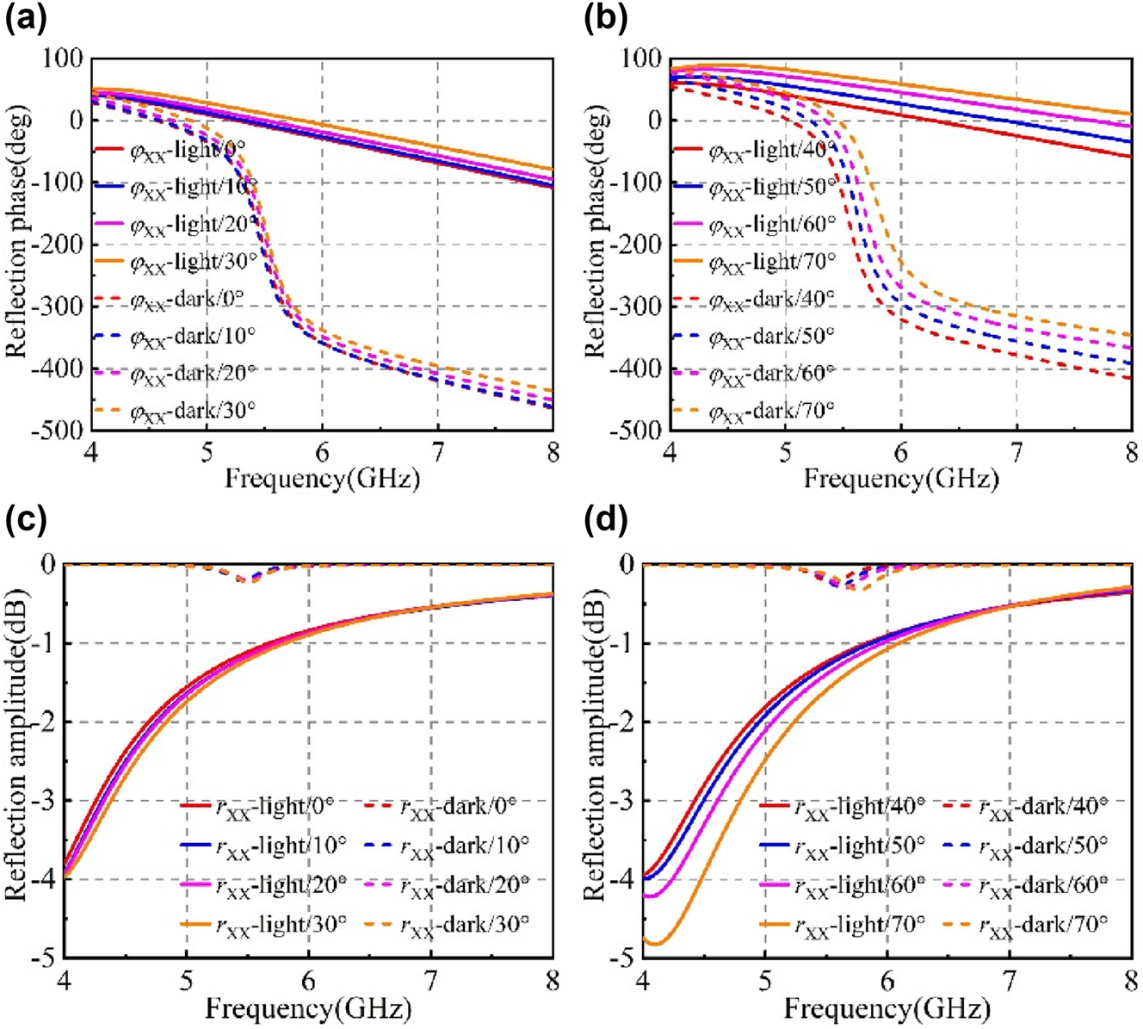
Under different incident angles: (a–b) reflection phase in the ‘0’ and ‘1’ states; (c–d) reflection amplitude in the ‘0’ and ‘1’ states.

In order to further verify that the metasurface has better application and robustness in practice, the EM response is simulated when the EM wave is incident at different angles, as shown in Figure 3. The reflection amplitude and phase of the meta-atom are shown in Figure 3a–d, respectively. When the incident wave is incident at different angles, the reflection phase difference at the resonant frequency is about π, and the reflection amplitude is almost equal, which meets the condition of phase modulation. With the incident angle increases, the resonant frequency gradually shifts to the right, from 5.47 GHz to 5.76 GHz. Therefore, the designed framework can also achieve reconfigurable characteristics under large incident angle.

## 3 Design and simulation

**Figure 4:**
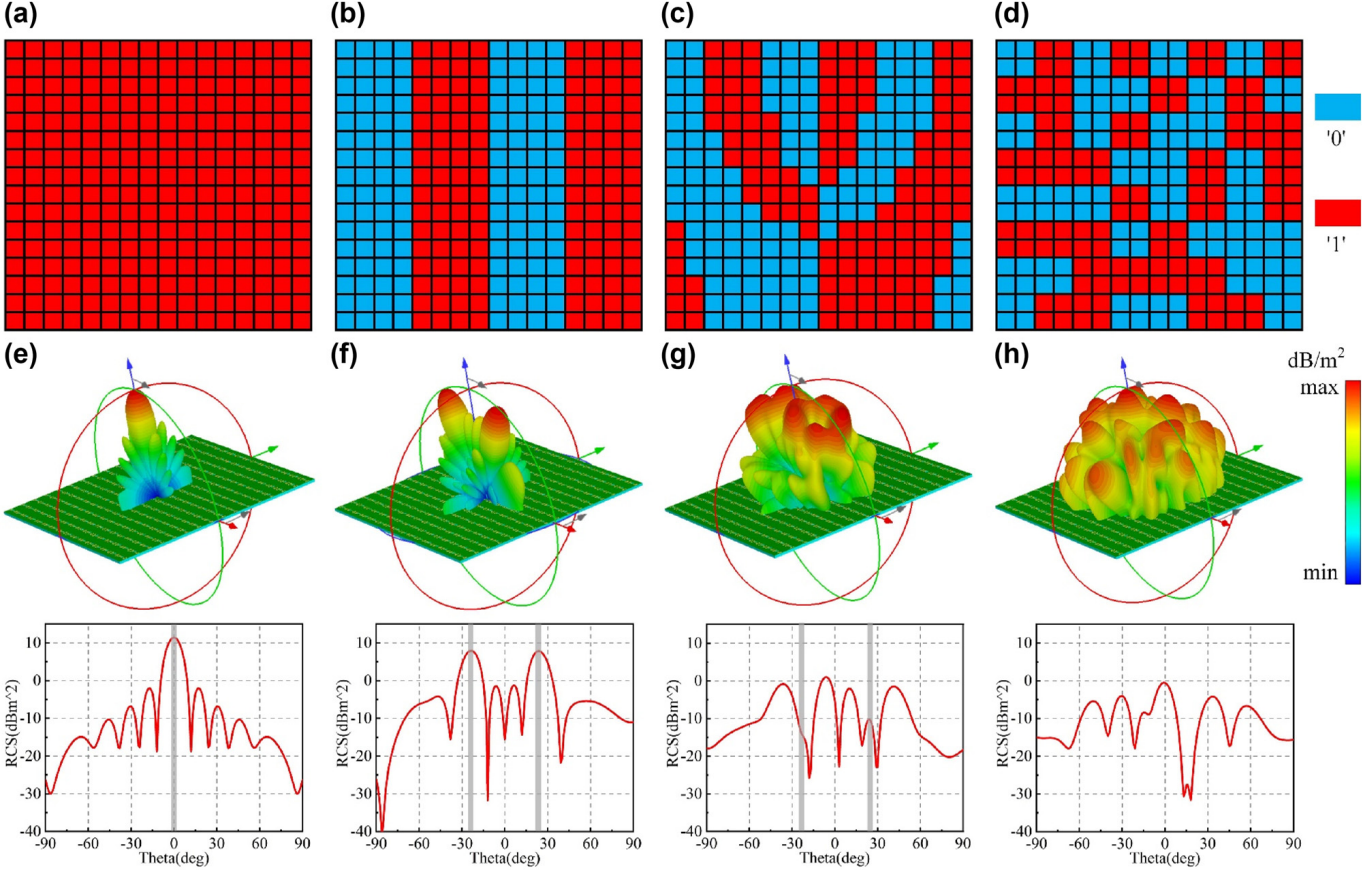
Simulation results of different EM functions: (a–d) coding sequence; (e–h) three-dimensional far-field scattering patterns and RCS curves.

According to the aforementioned design and analysis of the proposed reconfigurable meta-atom, a RM composed of 16×16 meta-atoms is constructed, whose phase coding sequence can be dynamically adjusted by the LED array. In this paper, the EM functions such as single beam, dual beam, vortex beam and RCS reduction are used to verify the feasibility and effectiveness of the design framework. Figure 4a–d shows the phase distribution of different EM functions, in which blue represents the code ‘0’ and red represents the code ‘1’. See Supplementary information Note S2 for more details on the calculation of the coding sequence. Three-dimensional far-field scattering patterns and RCS curves corresponding to different EM functions are shown in Figure 4e–h.

When the meta-atoms are in all ‘1’ states, a reflected beam along the main axis direction is generated, as shown in Figure 4e. When the codes ‘0’ and ‘1’ in the coding sequence are arranged alternately, the RM produces two obvious scattered beams, as shown in Figure 4f. The RM is encoded according to the coding sequence shown in Figure 4c, and the simulation result is shown in Figure 4g. It can be observed that the two beams generated have a spiral-shaped wavefront structure, showing a ring-shaped intensity distribution map with a hollow center, and the center nadir point of each beam is located near the pre-designed deflection angle, which is consistent with the characteristics of vortex beam. The RM is encoded according to the coding sequence shown in Figure 4d. The energy of reflected wave is well scattered in space, which can achieve the purpose of reducing RCS. Two-dimensional far-field scattering diagrams corresponding to different EM functions are shown in Figure S2(a-d). According to the simulation results, it is further proved that the metasurface can realize the reconfigurable characteristics in the microwave band. See Supplementary information Note S3 for more details on the two-dimensional far-field scattering pattern.

## 4 Experimental part

### 4.1 Reconfigurable characteristics test in the microwave band

**Figure 5:**
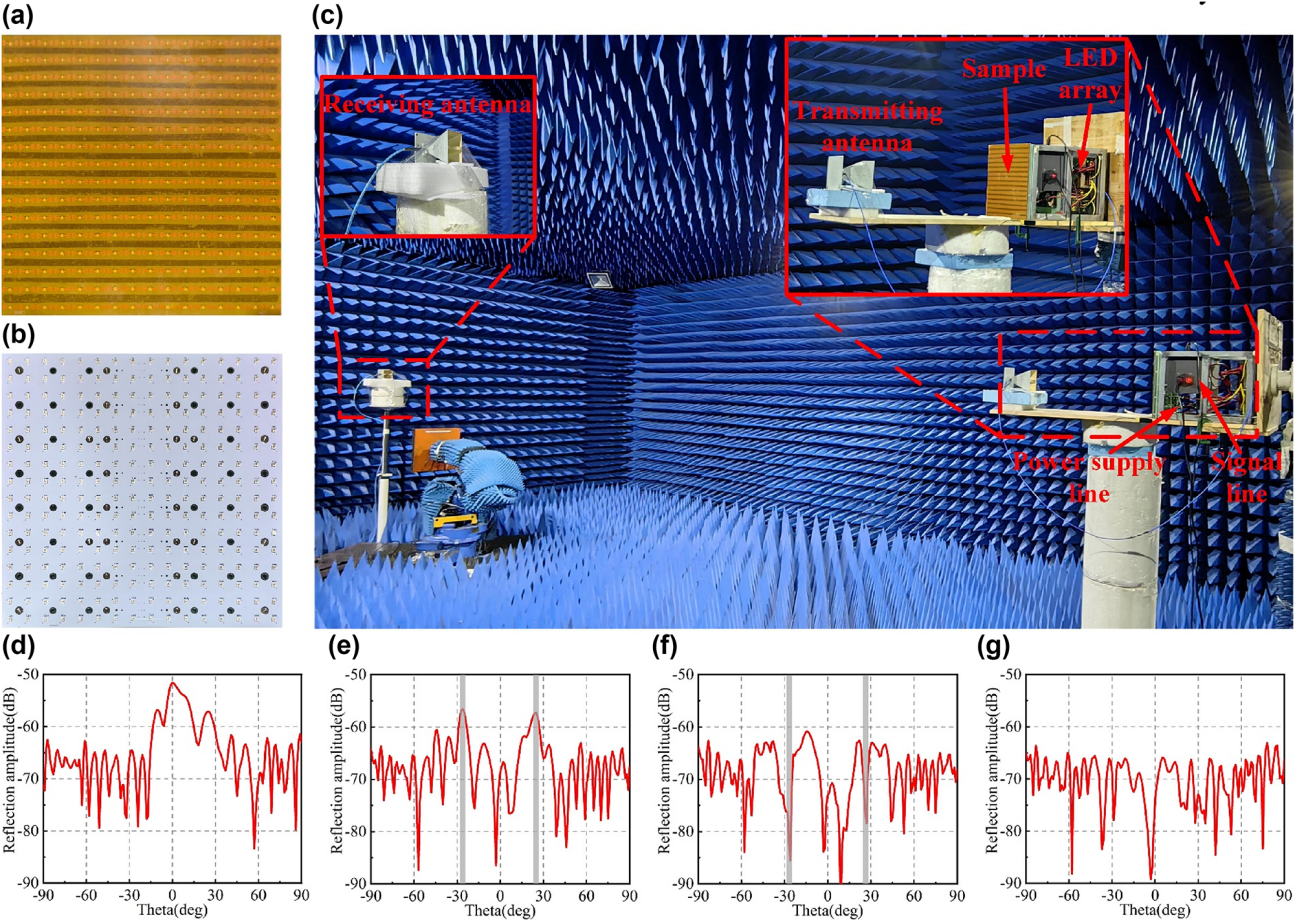
Experimental test in the microwave band: (a–b) sample prototype and LED array physical diagram; (c) schematic diagram of the experimental setup; (d–g) experimental results of different EM functions.

As shown in Figure 5a, an elaborate sample prototype identical to the simulation model is fabricated with low-cost printed circuit board (PCB) technology to further validate the practicability of designed RM, in which the required photoresistors are welded at the corresponding positions of the sample prototype. In order to excite the photoresistor, 2835 lamp beads are integrated on the aluminum substrate with integrated circuit packaging technology to make an LED array, as shown in Figure 5b. See Supplementary information Note S4 for more details on the photoresistor parameter test. The signal is transmitted to LED array through serial communication. Different coding sequences are converted into hexadecimal data frames by the computer. Further, the data frame is output to the LED array control board through the computer USB port and USB converter, which is used to control the on-off of each lamp and change the photoresistor value. See Supplementary information Note S5 for more details on the control method of the LED array.

In the microwave anechoic chamber, far-field test is performed on the prepared sample prototype. The experimental setup consists of the vector network analyzer (VNA), the linear polarized (LP) horn antenna, and the turntable mount, as exhibited in Figure 5c. In order to test the far-field scattering pattern of the sample prototype under x-polarized wave incidence, the transmitting horn antenna connected to VNA port 1 and the sample prototype are fixed on the turntable mount and rotated together, and the transmitting horn antenna is always placed along the sample prototype normal. The receiving horn antenna connected to VNA port 2 is placed far enough away from the sample prototype. At the beginning, two horn antennas are facing the processing sample prototype. According to the coding sequence shown in Figure 4a–d, the on-off of lamp beads is controlled by the computer to adjust the photoresistor value. The experimental results are shown in Figure 5d–g. When the meta-atoms are in the ‘1’ state, the incident EM wave is reflected back along the main axis, as shown in Figure 5d. According to the pattern of ‘0’ and ‘1’ alternately arranged, the RM can eliminate the main lobe beam between −90° and 90°, and produce two symmetrical beams. According to the coding sequence shown in Figure 4c, the RM generates two beams with hollow center between −90° and 90°, which accords with the characteristics of vortex beam. According to the random pattern, the reflected beam is less than −10 dB at all angles between −90° and 90°. Compared with Figure 4e–h, the experimental results are basically consistent with the simulation results, which verifies that the designed RM has reconfigurable characteristics in the microwave band.

### 4.2 Reconfigurable characteristics test in the infrared band

**Figure 6:**
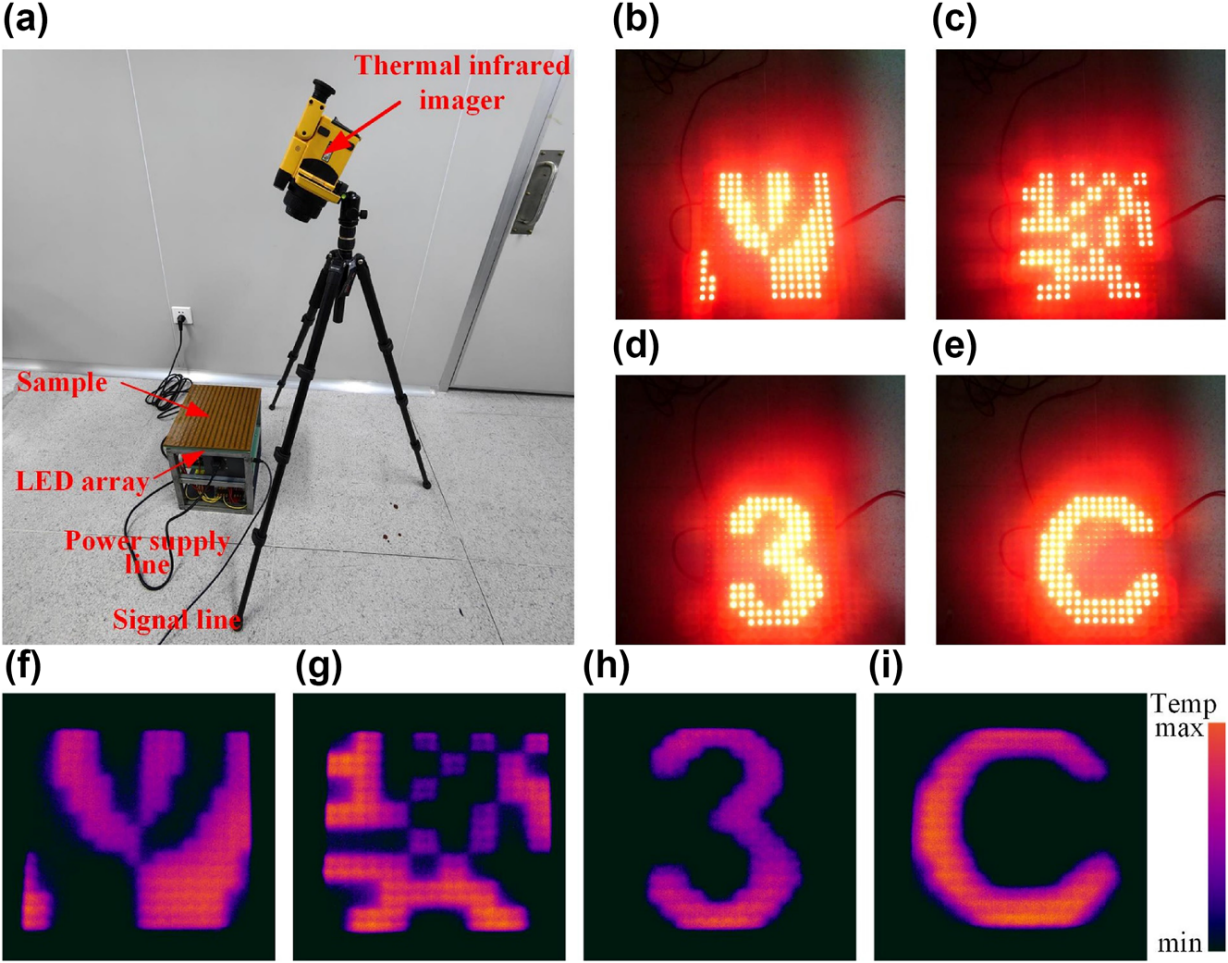
Experimental test in the infrared band: (a) schematic diagram of the experimental setup; (b–e) different luminous patterns; (f–i) infrared test results.

In this paper, the top layer material of RM is transparent PI film, and the emitted light can pass through the PI film, accompanied by the transmission of infrared light. The experimental setup is distributed as shown in Figure 6a. The frame, consisting of a metasurface and an LED array, is placed on the ground, connected to a power supply, and it is connected to a computer via a signal line to produce different luminous patterns. A thermal infrared imager is placed on a tripod to test the luminous condition. The vortex beam, RCS reduced, number ‘3’ and letter ‘C’ are selected for verification, as shown in Figure 6b–e. Different infrared emission conditions are tested through the thermal infrared imager. According to test results shown in Figure 6f–i, it can be verified that the designed framework has reconfigurable characteristics in the infrared band. The generated infrared pattern in Figure 6g shows completely different local intensities. This is mainly because the pattern size is inconsistent, which leads to the inconsistent temperature rise rate.

## 5 Conclusions

In this work, we propose a stimulator-multiplexing framework with reconfigurable characteristics in the microwave and infrared bands. The coding sequence is converted into hexadecimal data frames through computer to control the on-off of lamp beads. Based on this characteristic, the reflection phase can be adjusted to customize the far-field scattering mode. According to the experimental results, the RM realizes different EM functions through controlling the luminous situation of the LED array, which is basically consistent with the simulation results. This verifies that the designed framework has reconfigurable characteristics in the microwave band. In addition, the LED array excites the light source to result in the increase of temperature, which is accompanied by infrared characteristics. The different luminous condition of the LED array can be designed by the computer and tested by the thermal infrared imager. According to the test results, it can be verified that the framework has also reconfigurable characteristics in the infrared band. This work can realize the reconfigurable characteristics in the microwave and infrared bands with a single meta-atom structure, and it has potential application prospects in the fields of information transmission, and adaptive intelligent perception.

## Supplementary Material

Supplementary Material Details
