# Supplementary material for: Stimulator-multiplexing framework of microwave-infrared compatible reconfigurable metasurface integrated with LED array
Source: Nanophotonics. 2025 Mar 19;14(7):959–67. doi: 10.1515/nanoph-2025-0013 (PMC11980866; doi:10.1515/nanoph-2025-0013)
Supplement: Supplementary file 1 — Supplementary Material Details [file j_nanoph-2025-0013_suppl_001.docx]

Supporting Information

Stimulator-multiplexing Framework of Microwave-infrared Compatible Reconfigurable Metasurface Integrated with LED Array

Yuxi Li, Ruichao Zhu^*^, Sai Sui^*^, Yina Cui, Yuxiang Jia, Yajuan Han, Xinmin Fu, Cunqian Feng, Shaobo Qu, and Jiafu Wang^*^

**Note S1. Electromagnetic response of the meta-atom**


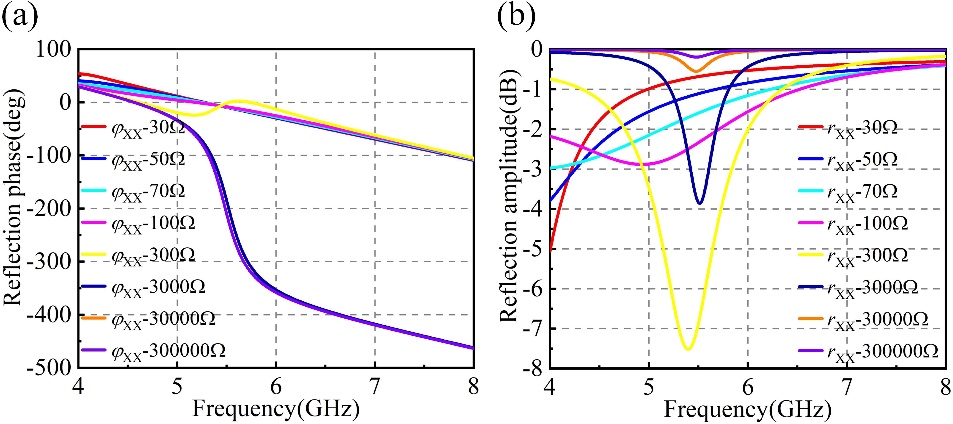


**Fig. S1.** (a) Reflection phase of the meta-atom under different resistance values; (b) Reflection amplitude of the meta-atom under different resistance values.

The electromagnetic (EM) response is simulated under different resistance values, as shown in Fig. S1. The reflection phase and amplitude of the meta-atom are shown in Fig. S1(a) and Fig. S1(b), respectively. According to Fig. S1(a), when the resistance value is 0.3MΩ, the reflected phase changes suddenly between 5-6GHz. In this range, when the resistance value is greater than 300Ω, there is a valley in the reflected amplitude. With the resistance value increases, the valley becomes shallower and shallower. When the resistance value is less than 100Ω, the valley disappears. With the resistance value decreases, the reflection amplitude curve gradually moves upward.

According to the simulation results, the reflection phase difference between 50Ω and 0.3MΩ is π, and the reflection amplitude is almost equal, which meets the conditions of phase modulation of the meta-atom.

**Note S2.** **Calculation of coding sequence**

**I. Multi-beam generation**

According to the generalized Snell’s law for the metasurface

(S1)

where $\theta_{r}$ is the reflection angle, $\theta_{i}$ is the incidence angle, $\lambda$ is the incidence wavelength, and is the phase gradient of the meta-atom. In this paper, the 1-bit code is used as an example for analysis. Assuming that the meta-atom side length is $d$ and each super meta-atom consists of $M\times M$ meta-atoms, then. Eq. (S1) is further simplified to

$\sin\theta_{r}-\sin\theta_{i}=\frac{\lambda}{2Md}$ (S2)

If the EM wave is incident perpendicular to the metasurface, the reflection angle of the EM wave can be calculated as

$\theta_{r}=\arcsin\frac{\lambda}{2Md}$ (S3)

get further

$M=\frac{\lambda}{2d\sin\theta_{r}}$ (S4)

According to Eq. (S3), it can be predicted that when $M=4$, the reflection angle is $\theta_{r}=25.37^{\circ}$. The coding sequence of dual beam is shown in Fig. 3(b).

**II. Vortex beam**

The reflection phase of the individual meta-atoms on the metasurface is calculated according to the reflection angle $\theta_{r}$

$\Phi(x,y)=l\arctan(\frac{y}{x})+k_{0}\sin(\theta_{r})x$ (S5)

where $l$ is the topology charge of the generated vortex beam, $k_{0}$ is the wave number in free space. Discretization of the reflected phase yields

$\Phi(x,y)=\left\{\begin{matrix} 0,0\leq\Phi(x,y)<180^{\circ} \\ 180^{\circ},180^{\circ}\leq\Phi(x,y)<360^{\circ} \end{matrix}\right.$ (S6)

With topological charge $l=\pm2$ and reflection angle $\theta_{r}=25^{\circ}$, the coding sequence of vortex beam is calculated according to Eq. (S5) and (S6), as shown in Fig. 3(c).

**III. RCS reduction**

If the reflected phases of the individual meta-atoms are randomly distributed, the metasurface can scatter the energy of the reflected wave, reduce the RCS caused by the metasurface, which can be given by Eq. (S7)

(S7)

Fig. 3(d) shows the RCS reduced coding sequence.

**Note S3. Two-dimensional far-field scattering pattern**

Two-dimensional far-field scattering diagrams corresponding to different electromagnetic functions are shown in Fig. S2(a-d).

As shown in Fig. S2(a), there is only one dark area in the image region, which clearly indicates that the metasurface generates a directional reflected beam along the principal axis. In Fig. S2(b), two distinct dark regions appear along the x-axis, indicating that the metasurface disperses the incident EM wave into two scattered beams in different directions. As shown in Fig. S2(c), the phase rotates around the center along the azimuth direction. This feature shows that the metasurface successfully produces vortex beam. As shown in Fig. S2(d), there are several dark regions randomly distributed throughout the image area, indicating that the metasurface scatters EM wave in all directions in space.


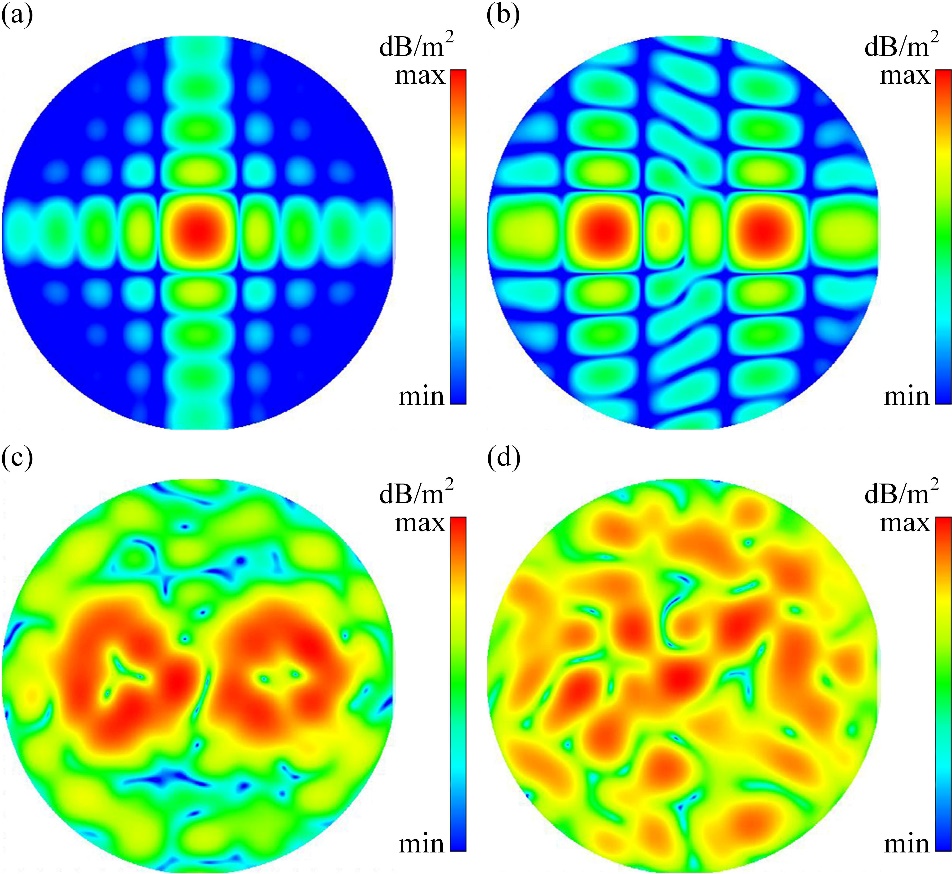


**Fig. S2.** Two-dimensional far-field scattering pattern of different EM functions: (a) single beam; (b) dual beam; (c) vortex beam; (d) RCS reduction.

**Note S4.** **Photoresistor parameter test**

In this paper, GT36516 photoresistor is selected as the active device of the meta-atom. When the light source irradiate the photosensitive surface of the photoresistor, the resistance value gradually drops to tens of ohms and reaches a stable state after 20ms. When the light source disappears, the resistance value rises and returns to the initial value 0.3MΩ after 30ms. When the resistance reaches a stable state in the light environment, its resistance value is almost unchanged. In order to excite photoresistors, an LED array with 2835 lamp beads is designed. Next, the photoresistor value under different wavelength light sources is measured and it is determined which wavelength the resistance is sensitive to. In this paper, the 2835 lamp beads with the wavelengths of 520nm, 560nm, 590nm, and 620nm are selected. The luminous effect of the lamp bead is shown in Fig. S3.

The different wavelengths lamp beads are placed at different distances above the photoresistor and the multimeter is used to measure the photoresistor value, as shown in Tab. S1. According to Tab. S1, the longer the wavelength and the closer the distance, the smaller the photoresistor value. It can also be obtained that the photoresistor is more sensitive to lamp beads with a wavelength of 620nm. In this condition, when the distance is 10cm, the resistance value is about 50Ω, which meets the prerequisites for regulating metasurfaces.


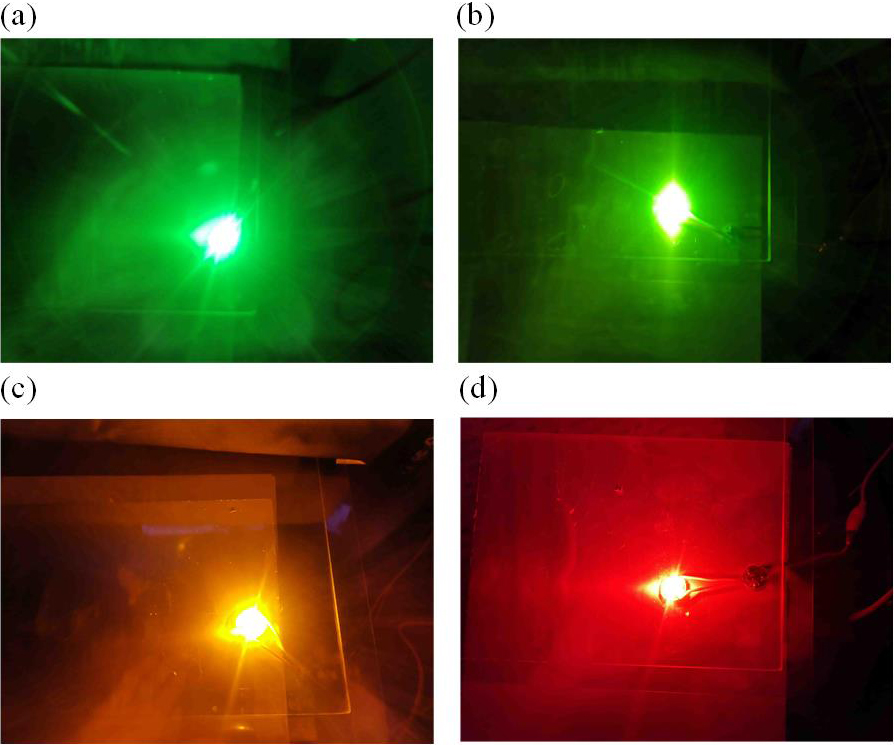


**Fig. S3.** 2835 lamp bead test: (a) Luminous effect of 520nm wavelength lamp bead; (b) Luminous effect of 560nm wavelength lamp bead; (c) Luminous effect of 590nm wavelength lamp bead; (d) Luminous effect of 620nm wavelength lamp bead.

**Tab. S1.** Photoresistor value in different situations

| Distance(mm) | | 3mm | 4mm | 5mm | 10mm | 15mm |
| --- | --- | --- | --- | --- | --- | --- |
| 520nm | Light intensity(lx) | 110000 | 97000 | 88000 | 43000 | 25000 |
|  | Resistance(Ω) | 148 | 153 | 160 | 244 | 297 |
| 560nm | Light intensity(lx) | 75500 | 66000 | 60000 | 27000 | 17000 |
|  | Resistance(Ω) | 92 | 102 | 110 | 167 | 216 |
| 590nm | Light intensity(lx) | 56500 | 36000 | 30000 | 22000 | 11100 |
|  | Resistance(Ω) | 70 | 82 | 90 | 118 | 180 |
| 620nm | Light intensity(lx) | 88000 | 85000 | 83000 | 50000 | 26000 |
|  | Resistance(Ω) | 49 | 56 | 65 | 84 | 113 |

**Note S5.** **Control method of LED array**

The LED array control system consists of a computer, USB converter, LED array control board, and power supply, as shown in Fig. S4. The control system is planned by the computer as the host computer. Through the computer USB port and USB converter, the control data frame is output to the control board to control the brightness of the lamp beads on the LED array. Based on this characteristic, it is possible to adjust the photoresistor value. Data transfer between the computer and the USB converter is achieved through the analog serial port in the computer.


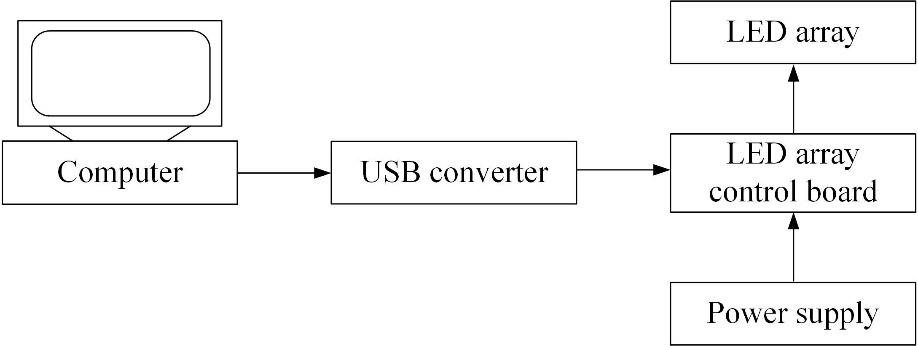


**Fig. S4.** LED array control system

There are 256 photoresistors welded on the metasurface, so it is necessary to integrate 256 lamp beads on the LED array. The naming rules are from left to right and from top to bottom on the front of the array, and $16\times16$ lamp beads on the LED array are named as numbers 1-256 in turn. The specific naming situation is shown in the Fig. S5.

The status information of the lamp bead can be represented by binary coding, with '1' indicating that the lamp bead is on and '0' indicating that the lamp bead is off. According to the order of naming numbers 1-256, every 8 numbers make up a byte, which can make up 32 bytes in total. In each byte, the small named number is the high bit of the byte, and the large named number is the low bit of the byte. In this programming control mode, as long as each byte and the bit in the byte are determined, the on-off state of the lamp bead can be controlled. These information and two bytes of the length of the flag information constitute a data frame sent to the USB converter. Those information and two bytes of flag information length form a data frame sent to the USB converter. For such a data frame in our current application, it contains 34 bytes, of which the first two bytes indicate the length of the following data. Because the LED array in this paper needs to use 18 bytes, the data length here is 0x20. According to the coding sequence, the data frame is edited and sent to the LED array. The editing of information and the initiation of transmission need to be done in the computer. Different data frames represent different coding methods, and different lighting patterns are designed through computer programming. Based on this characteristic, the designed framework in this paper can dynamically control EM waves, change the reflection phase and customize the far-field radiation mode.


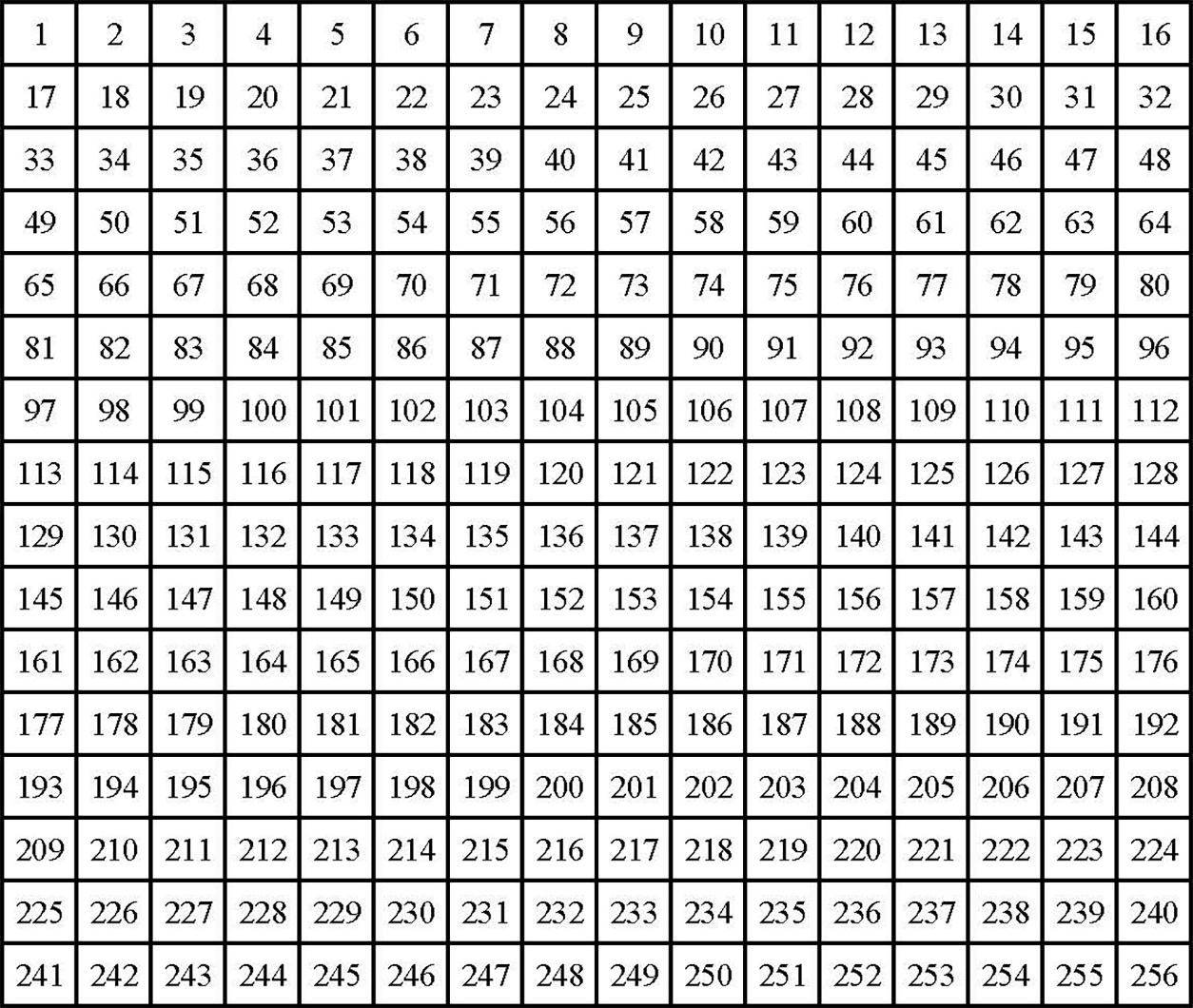


**Fig. S5.** The named number of the lamp beads on the LED array
